# Multi-targeted management of upland game birds at the agroecosystem interface in midwestern North America

**DOI:** 10.1371/journal.pone.0230735

**Published:** 2020-04-27

**Authors:** Marlis R. Douglas, Whitney J. B. Anthonysamy, Steven M. Mussmann, Mark A. Davis, Wade Louis, Michael E. Douglas

**Affiliations:** 1 Biological Sciences, University of Arkansas, Fayetteville, Arkansas, United States of America; 2 Illinois Natural History Survey, University of Illinois, Champaign, Illinois, United States of America; 3 Illinois Department of Natural Resources, Gibson City, Illinois, United States of America; University of Regina, CANADA

## Abstract

Despite its imperative, biodiversity conservation is chronically underfunded, a deficiency that often forces management agencies to prioritize. Single-species recovery thus becomes a focus (often with socio-political implications), whereas a more economical approach would be the transition to multi-targeted management (= MTM). This challenge is best represented in Midwestern North America where biodiversity has been impacted by 300+ years of chronic anthropogenic disturbance such that native tall-grass prairie is now supplanted by an agroecosystem. Here, we develop an MTM with a population genetic metric to collaboratively manage three Illinois upland gamebirds: common pheasant (*Phasianus colchicus;* pheasant), northern bobwhite quail (*Colinus virginianus*; quail), and threatened-endangered (T&E) greater prairie chicken (*Tympanuchus cupido pinnatus*; prairie chicken). We first genotyped our study pheasant at 19 microsatellite DNA loci and identified three captive breeding stocks (N = 143; IL Department of Natural Resources) as being significantly bottlenecked, with relatedness >1^st^-cousin (μR = 0.158). ‘Wild’ (non-stocked) pheasant [N = 543; 14 Pheasant-Habitat-Areas (PHAs)] were also bottlenecked, significantly interrelated (μR = 0.150) and differentiated (μ*F*_ST_ = 0.047), yet distinct from propagation stock. PHAs that encompassed significantly with larger areas also reflected greater effective population sizes (μ*N*_E_ = 43; P<0.007). We juxtaposed these data against previously published results for prairie chicken and quail, and found population genetic structure driven by drift, habitat/climate impacts, and gender-biased selection via hunter-harvest. Each species (hunter-harvested or T&E) is independently managed, yet their composite population genetic baseline provides the quantitative criteria needed for an upland game bird MTM. Its implementation would require agricultural plots to be rehabilitated/reclaimed using a land-sharing/sparing portfolio that differs markedly from the Conservation Reserve Program (CRP), where sequestered land decreases as agricultural prices escalate. Cost-savings for an MTM would accrue by synchronizing single-species management with a dwindling hunter-harvest program, and by eliminating propagation/stocking programs. This would sustain not only native grasslands and their resident species, but also accelerate conservation at the wildlife-agroecosystem interface.

## Introduction

Anthropogenic impacts are a serious challenge for biological diversity [1], with major contributors being global climate change and habitat fragmentation. Each can rapidly and independently extirpate biodiversity (i.e., "with high confidence [2]," [3,4]). Recreational hunting also exerts a relatively consistent pressure [5], under the tenet that wildlife is a resource that can be optimally harvested, much like timber, yet with similar complications [6].

Interestingly, the interactions among anthropogenic drivers has been relatively unexplored [7], due largely to difficulties in gauging their gradual, consistent, and low intensity impacts in the field. However, reliable quantification has occurred in the laboratory. The synergy among overharvest, habitat fragmentation, and environmental warming, for example, reduces rotifer populations 50x faster than each driver acting independently [8]. This issue was first recognized some 30 years ago [9], with impacts identified as multiplicative rather than additive. Yet surprisingly, subsequent research has been limited [10,11], and perceptions have shifted concomitantly. A more expansive interpretation now recognizes this synergy as “… a chronic anthropogenic disturbance” [12,13] strongly associated with global species-extinctions (25% mammalian, 13% avian, and >21,000 ‘other;’ [14]).

### Multi-species management

The gradual but ongoing progression of chronic anthropogenic disturbance negatively impacts adaptive management, and in a variety of ways. For example, it seriously strains budgets [15], and its gradual manifestation often promotes managerial indecision. In addition, studies that strive to quantify its effects often yield results that vary temporally [16], and with ambiguous interpretations. More traditional conservation programs attempt to compensate by shifting perspectives from local to regional [17], yet this often creates difficulties in that a coalesced approach can be too broad of a template for single-species management [18].

In contrast, an MTM approach (i.e., a framework for management decisions with benefits optimized for a community) offers several positive considerations. It can reduce management conflicts when several at-risk species are incorporated, and adequately address common threats across each while simultaneously eliminating the potential for species-specific redundancy [19]. Conservation efforts can also be effectively optimized under this approach, particularly when study species are taxonomically related, co-occur in similar habitats, and are impacted by comparable threats (as herein). We employed these considerations in this study as a focus for our management plan.

### Midwestern North America

A history of chronic anthropogenic disturbance is clearly reflected in midwestern North America, with its 300+ year record of forests felled, prairies plowed, and streams sequestered for agricultural and urban purposes. The region supports an energy-intensive economy where greenhouse gas emissions exceed the national average by >20% [20]. Furthermore, its growing season and agricultural row-crop technology have now been significantly extended [21, Table 1], and both greatly enhance the well-established agricultural capacity of the region [22].

**Table 1:** Genetic diversity estimates based on 19 microsatellite DNA loci derived for 686 pheasant sampled from 14 Illinois pheasant habitat areas (PHAs; see S1 Table for abbreviations).

PHA	ha	*N*	Av*A*	Av*H*e	R	sd	*N*e	Lower	Upper	Wilcox
CHGF	40.7	25	5.32	0.59	0.123	0.019	32.1	24.1	45.6	**0.036**
DOHB	35.6	24	4.79	0.61	0.157	0.023	19.1	14.9	25.3	**0.001**
DWBB	32.4	28	5.11	0.56	0.147	0.021	35.9	26.8	51.6	0.052
DWFF	148	68	5.63	0.57	0.166	0.023	25.0	22.3	28.1	**0.006**
DWHV	33.6	26	4.79	0.52	0.24	0.029	45.8	30.9	79.9	0.430
FOSI	255	96	6.00	0.58	0.166	0.033	80.7	68.9	95.9	**0.002**
IQCF	32.0	26	4.79	0.56	0.166	0.033	14.2	11.5	17.7	**0.004**
IQLO	64.8	26	5.68	0.59	0.129	0.02	47.4	34.0	73.9	**0.048**
IQMG	30.8	51	6.63	0.60	0.122	0.018	23.7	21.3	26.5	**0.044**
KXVI	208	24	4.95	0.56	0.156	0.019	83.2	45.6	314.4	**0.019**
LEST	32.4	23	4.58	0.55	0.181	0.025	21.0	15.7	29.7	**0.048**
MLSB	261	85	6.05	0.59	0.113	0.014	90.3	75.4	110.7	**0.003**
SKBD	41.7	25	5.32	0.59	0.142	0.018	50.0	34.1	86.1	**0.040**
VEHW	57.1	16	5.16	0.63	0.092	0.016	37.6	23.2	82.0	**0.007**
Mean	83.8	39	5.34	0.58	0.150		43.3			
JHGF	n/a	67	6.58	0.61	0.101	0.011	210.0	150.6	334.3	**0.020**
JHMA	n/a	43	7.00	0.65	0.143	0.012	110.0	80.8	165.9	**0.001**
MFMA	n/a	33	6.26	0.61	0.23	0.014	60.5	45.4	86.8	**0.033**
Mean	n/a	48	6.61	0.62	0.158		126.8			

Bottom three rows represent propagation stocks, designated as: JHGF = J. Helfrich “game farm;” JHMA = J. Helfrich Manchurian; and MFMA = MacFarlane Manchurian. Headers are: ha = area in hectares; *N* = number of individuals; Av*A* = mean number of alleles per locus; Av*H*e = average estimated heterozygosity; R = relatedness; sd = standard deviation of R; *N*e = effective population size; Lower = lower confidence interval of Ne; Upper = upper confidence interval of *N*e; Wilcox = Wilcoxson rank-sum estimate for bottleneck (bold values are statistically significant).

Such enhancements, while economically positive, have also compressed regional biodiversity into novel prairie-like parcels distributed randomly across an expansive agricultural matrix. Its persistence is inexorably challenged not only by a suite of ongoing anthropogenic pressures (as above), but also by a concomitant erosion of ecosystem services (i.e., anthropogenic benefits directly or indirectly received; [23]). Yet the negative aspects of agriculture, as seen from a conservation stance [24], may in fact offer positive considerations when placed within a more management-oriented land-sharing/sparing portfolio (below).

### Upland game birds

Upland birds are an historic component of North American biodiversity, well documented in both Pleistocene fossil records and the earliest regional ornithological collections [25]. Their life histories juxtapose with the extensive open grassland on the east coast of North America, as promoted by natural disturbances such as wildfire and the felling of trees by beaver. The burning and clearing practices of Native Americans sustained and extended open areas and were subsequently emulated by early European settlers [25].

Chronic anthropogenic disturbance had an early initiation in North America, and subsequently became quite challenging for upland game birds, many of which are (or were) hunter-harvested [26]. We now have a management imperative to sustain these species, not just from an economic stance [27], but also in support of a uniquely American tradition: hunting open to all but subject to access when land is privately owned [28]. An additional challenge is that federal/state agencies are often tasked with dual but diametrically opposed mandates in this regard: to accommodate wildlife for recreation on one hand, yet also sustain and recover T&E species on the other. Thus, an ongoing requirement is to gather sufficient data for congruent, economically feasible management strategies that extend across multiple species. Contemporary technologies and integrated approaches such as a land-sharing/ land-sparing portfolio help facilitate decision-making and stand in contrast with more politically-biased policies [29] such as The Conservation Reserve Program (CRP, a provision of the 1985 U.S. Farm Bill) (discussed below).

### Genetic integration and MTM

The spatial constraints of habitat fragmentation generally require management at the population genetic level, in that genetic drift (i.e., random fluctuations in allele frequencies over time; [30]) is a frequent byproduct. A second important parameter is effective population size (*N*_e_), which reflects the loss of heterozygosity due to drift and links strongly with demographic factors such as sex ratio, population size, and lifetime fitness. Consequently, those population effects manifested through demography and environment can best be gauged by evaluating *N*_e_.

In a similar manner, severe impacts also emerge when the size of a population is reduced by harvest. In the near term, genetic variability and individual fitness are depleted, with the trajectory of the population being depressed in the long-term [31]. Ongoing selection on gender and maturity also targets the reproductive component of populations, with reverberations again tracked via *N*_e_. Importantly, these effects can be not only documented with population genetic tools, but also remediated as well [32]. Despite these caveats, population genetic approaches have yet to be fully implemented into wildlife management [32,33], as opposed to that found in fisheries [34].

**Fig 1:**
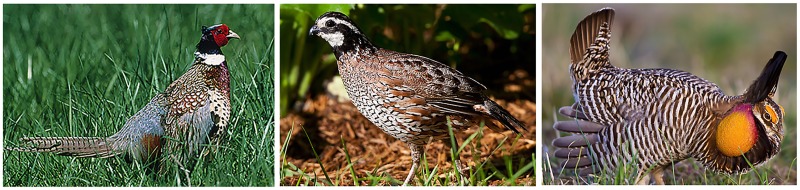
Study species. (A) Male common pheasant, Jefferson Co., IL; (B) Male greater prairie chicken on booming ground, Jasper Co., IL; (C) Male northern bobwhite quail, Marion Co., IL (pictures courtesy of Richard Day, Daybreak Imagery, Alma IL 62807, www.daybreakimagery.com/).

We employ population genetic approaches to characterized genetic diversity as it relates to chronic anthropogenic disturbance in the non-native common pheasant [(*Phasianus colchicus*; pheasant)] [35]. We then contrasted these results with those previously derived for the state-endangered greater prairie chicken (*Tympanuchus cupido pinnatus*; prairie chicken) [36] and hunter-harvested northern bobwhite quail (*Colinus virginianus*; quail) [37] (Fig 1).

In this sense, our comparative approach extends from introduced to native species, and from hunter-harvested to T&E components. This allows us to explore the capacity of population genetics as a baseline for an upland game bird MTM. Our contemporary and economical approaches were designed to resonate with stakeholders and interest groups [38]. By doing so, we transition policy and planning from a more traditional single-species approach to one that engages multiple species [32]. We first evaluated wild pheasant in Illinois, as sampled from non-supplemented habitat fragments (PHAs: pheasant-habitat-areas). We then tested if these were distinct from state-maintained propagation stock employed annually to supplement “controlled hunting reserves” (CHRs). We then tested PHAs for temporal and/or spatial structure, and for evidence of inbreeding and interrelatedness. These results were contrasted with those from two other Illinois upland game species (i.e., prairie chicken and quail). This allowed us to ascertain whether our composite results are a basis for a state-driven MTM plan, and a potential blueprint for a similar plan region-wide.

## Materials and methods

### Upland game bird natural history

#### Common pheasant

Pheasant was initially introduced into Oregon from mainland China (1880–81; available from: https://www.orvis.com/s/upland-game-birds-of-north-america/14692) to serve as an additional upland game bird suitable for anthropogenic hunter-harvest. However, repeated serial releases by state and federal agencies were required over many years before it became a self-sustaining component of the Great Plains and an icon of hunter-harvest [39]. Its abundance increased steadily through the mid-20th century, peaking in the early 1960s with >one million harvested [40]. Hunter-harvest subsequently declined continent-wide through the 1970s, with <30,000 harvested in Illinois during 2016 [41].

**Fig 2:**
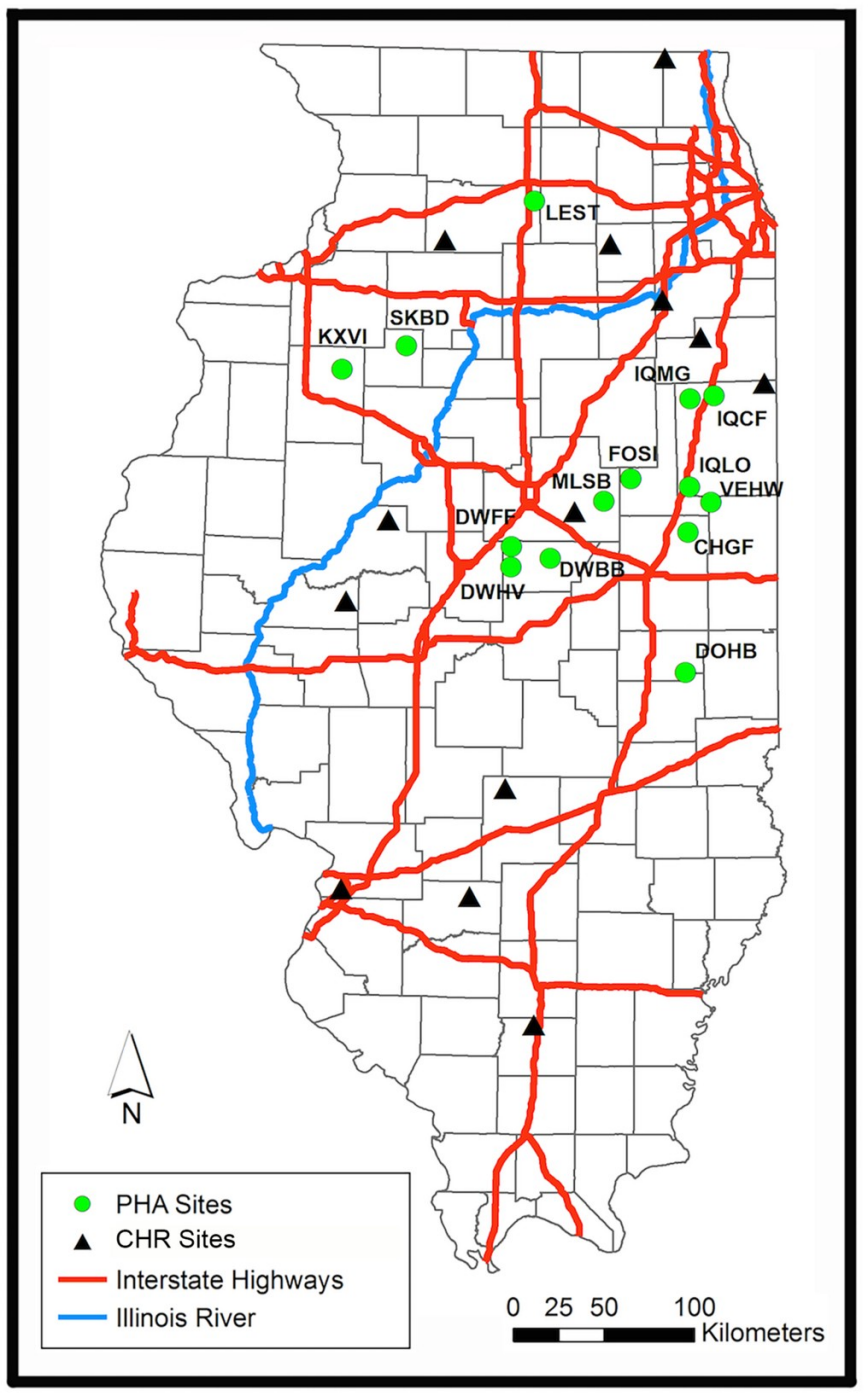
Map of Illinois depicting pheasant habitat areas (PHAs) and controlled hunting reserves (CHRs). Interstate highways (= red); Illinois River (= blue); non-supplemented Illinois Department of Natural Resources (IDNR) Pheasant Habitat Areas (PHAs = green dots; N = 14); Controlled Hunting Sites (CHRs = black triangles; N = 13) are supplemented by propagated pheasant.

The Illinois Department of Natural Resources (IDNR) proactively established 22 statewide Pheasant Habitat Areas (PHAs) as a non-augmented public hunting resource, with male-only take allocated via lottery. Additional ‘controlled hunting reserves’ (CHRs) also provide recreational opportunities, supplemented annually by state-propagated stock. The potential for gene flow among PHAs and CHRs is reduced by intervening agricultural land (20km minimum; [42]) (Figs 1 and 2).

Small, temporary groups of male pheasant coalesce during winter, whereas females represent larger, more stable flocks. Prominent components of pheasant life history are territorial defense and polygyny, with males actively competing for and subsequently defending territories. Here, the male strategy is to actively monopolize open ground adjacent to cover, as these represent prime locations for females to forage [43]. The latter disperse in spring and are actively recruited by males into small harems within territories. Females will nest outside these territories yet often return to the same male the following year.

#### Greater prairie chicken

Much like pheasant, the prairie chicken is also a ground-nesting game bird that inhabits mixed-grass/ tallgrass prairie interspersed with patches of cropland. Dense brush is critical for nesting, as it offers protection from climate and predators, whereas more open areas are necessary for foraging. During winter, prairie chicken gather near croplands to access supplemental food. However, modern agricultural techniques frequently reduce and fragment adjoining habitat, and this leads to sharp declines in population numbers (available from: https://www.allaboutbirds.org/guide/Greater_Prairie-Chicken/lifehistory).

The breeding strategy of prairie chicken involves dominance polygyny, where males display for females on leks. Yet only a small subset subsequently reproduce. Prairie chicken was once widely distributed across the North American great plains but has now been reduced to small, isolated fragments that require intensive management. It has declined sharply in Illinois from millions (mid-nineteenth century), to 2000 (1962), then 46 (1998), necessitating serial translocations from out-of-state. The most recent four-year population estimate (2010–2013; [36]) averaged but 79 males.

#### Northern bob-white quail

This species resides year-round in ephemeral upland habitat with multiple successional stages, to include agricultural fields, grasslands, and open forest. It exhibits many characteristics of an r-selected species, i.e., early reproduction, high reproductive capacity, and short life span [37]. Bobwhite is highly social, often found in groups, or coveys containing up to 20 individuals that roost in a close-packed, outward-facing circle so as to conserve heat and sustain group-awareness. It was once common in eastern North America, but now reflects substantial widespread and cumulative declines (~85%; available from: https://www.allaboutbirds.org/guide/Northern_Bobwhite/lifehistory).

Small-scale agriculture often provided suitable habitat for quail, but this has largely been eliminated by the advent of larger, more mechanized farming practices. For example, old fields were once prime habitat for quail. They have not only been replaced, but those remaining have been invaded by exotic grasses that now render them unsuitable for quail. In addition, larger tracts of intensive row crop agriculture, or contiguous mature forest, now act to segregate quail populations and promote genetic drift.

### Pheasant samples and DNA techniques

From 2010–2012, feathers from wild males (= ILWI) were harvested by lottery-selected hunters across 22 PHAs (Fig 2), thus no IACUC approval was required. Feathers were also obtained from three propagated stocks: (1) a private facility as a source of original Manchurian stock (MacFarlane Pheasants, Inc., Janesville, Wisconsin = MFMA) < available from: https://www.pheasant.com/>; (2) original Manchurian stock now maintained for many years by IDNR (James Helfrich Wildlife Propagation Center, Lincoln, Illinois = JHMA); and (3) ‘game farm’ progeny derived from JHMA roosters x ILWI hens (Helfrich Propagation Center = JHGF). The latter are used to restock CHRs on an annual basis. We extracted genomic DNA from sample feathers using a protocol (Qiagen DNeasy^®^ Kit) that compensates for low DNA yields.

#### Microsatellite amplification and genotyping

We tested 83 microsatellite DNA loci originally developed for eight galliform species [44,45,46,47,48,49,50], with 24 (29%) yielding unambiguous genotypes. Forward primers were labeled with Applied Biosystems (ABI) fluorescent dyes. Polymerase chain reactions (PCR) were run in 10–15μl volumes containing 1x Go-*taq* flexi buffer (PROMEGA), 3.5mM MgCL_2_, 0.25mM dNTPs, 0.2μg BSA, 0.5–1.0 units Go-*taq* DNA polymerase (PROMEGA) and 40ng DNA. Cycling conditions were: initial denaturation 3m at 95°C, 15 cycles for 45s at 95°C, 45s at 52°C, 1m at 72°C, 25 cycles for 30s at 95°C, 30s at 52°C and 45s at 72°C. Fragments were resolved on an ABI 3730 Genetic Analyzer and an internal size standard (Liz500) was included with each sample.

Alleles were scored with GeneMapper v4.0 (ABI) and data quality assessed for three captive stocks and each of the 14 PHAs using MicroChecker v2.2.3 [51]. Deviations from Hardy–Weinberg Equilibrium (HWE) and linkage equilibria (LD) were computed using exact tests in GenePop [52,53], with *P*-values estimated via Markov Chain with 10,000 dememorizations, 200 batches, and 5,000 iterations. Level of significance was evaluated using sequential Bonferroni tests.

#### Genetic diversity and population structure

Genotypes were assayed across 19 microsatellite DNA loci. From these data, standard genetic indices were calculated, including observed heterozygosity (*H*_O_) and mean number of alleles (A_M_) for each of 14 PHAs N = 543) and captive propagation stock (i.e., MFMA, JHMA, JHGF; N = 143) (GenAlex v6.5 [54]). *H*_O_ is proportional to the amount of genetic variance at a microsatellite locus (i.e. heritability), yet also reflects the manner by which genetic variation is impacted by population size. Allelic richness (A_R_) and private allelic richness (A_PR_) was estimated using rarefaction based on the smallest diploid sample (HP-Rare [55]).

An hierarchical approach was employed to: (1) evaluate genetic diversity and divergence among wild pheasant (PHAs) and propagation stocks (i.e. MFMA, JHMA, JHGF), and (2) assess population genetic structure among PHAs. Pairwise *F*_ST_ values were calculated to assess gene flow among the four groups, as well as among PHAs (Arlequin v3.5 [57]). To gauge isolation-by-distance (IBD) among PHAs, a Mantel test was employed (GenAlex) so as to compare pairwise genetic distance [*F*_ST_/ (1- *F*_ST_)] and pairwise geographic distance (log_10_+1 transformed).

A Bayesian assignment test (Structure v2.3.4 [58]) was employed to assess genetic structure among propagated stocks and PHAs. The combined analysis involved an admixture model with no priors and correlated allele frequencies, with K-values = 1–20. The program was run for 1,100,000 generations with the first 100,000 discarded as burn-in. Independent replicates (N = 32) were performed at each K-value to test for consistency and to derive ΔK and L(K) [59]. As recommended [61], ΔK was compared against biological and biogeographic patterns (Structure Harvester v0.6.94 [60]), with both statistics evaluated to determine an appropriate K [61]. Outputs were assessed (Clumpp v1.1.2 [62]) to ascertain multimodality at each K, with individuals then appropriately assigned to gene pools (Distruct v1.1 [63]). The above process was repeated for PHAs only, using a more restricted range of K values (K = 1–15), with similar iterations, burn-in, ΔK derivation, and visualizations as above.

#### Demography and consanguinity

Population persistence can be gauged in several ways, but most frequently via demographics (i.e., small population paradigm [64]). Here we quantified recent population bottlenecks (<five generations) by contrasting *H*_*o*_ empirically derived against that expected (*H*_*e*_) under Hardy–Weinberg equilibrium (HWE) (BottleNeck 1.2.02 [65]).

We applied the infinite alleles model (IAM [66]) to gauge significance of heterozygosity-excess in each PHA (Wilcoxon signed-rank test [67]). A mode-shift test was also applied to evaluate historic bottlenecks (i.e., ~20 generations), with *N*_e_ (heterozygosity loss in each generation due to genetic drift) estimated for each PHA and propagation stock using the linkage disequilibrium (LD) method (NeEstimator_v2 [68]). Estimates were derived at *P* = <0.01, with jackknifed 95% confidence intervals.

BayesAss3 [56] was employed to test for demographic independence among propagation stocks and PHAs. A Bayesian approach (NewHybrids [69] (https://github.com/eriqande/newhybrids) was used to examine hybridization/introgression between Manchurian, game farm, and PHAs. Individuals were assigned via posterior probabilities into one of six hybrid classes (i.e., pure game farm; pure Manchurian; F_1_; F_2_; game farm backcross; Manchurian backcross) [70]. All Manchurians were classified as one parental and game farm as a second. Calculations were performed with 100,000 burn-in generations followed by 1,000,000 sampling generations.

Mean relatedness (*R*) was used to test for siblings and/or parent–offspring pairs (TrioML method; Coancestry v1.0 [71,72]). A value of *R* = 0 indicates no relation; *R* = 0.125 = 1^st^ cousin, *R* = 0.25 = half-siblings, and *R* = 0.5 = parent/offspring or full sibling [73]. A Mantel test in GenAlex contrasted pairwise mean relatedness *versus* geographic distances separating PHAs.

#### Spatial structure

Genetic discontinuities were evaluated by using a Bayesian clustering method (R-package Geneland ver. 4.0.4 [74]) to model the multi-locus, geo-referenced pheasant genotypes. The uncorrelated model of allele frequencies was utilized, as was a non-zero value for the uncertainty among coordinates. This allowed the program to assign individuals to different clusters despite being sampled from the same site. It also has the potential to detect migrants that might otherwise remain undiscovered by taking into account their spatial coordinates, then allocating genotypes into K-clusters with HWE and LD minimized within groups. Four independent runs with 10 million MCMC iterations were performed, with every 1,000^th^ being saved. The number of genetic clusters (K) was initially set to vary between 1 and 21, and the model was run four consecutive times, with clusters treated as ‘known’ based upon inferences from previous runs. The posterior probability of population membership was computed using a 300-iteration burn-in. Spatial relationship among PHAs was also evaluated by testing for isolation-by-distance (IBD).

We performed a Discriminant Analysis of Principal Components (DAPC) to visualize differentiation among PHAs and propagation stock (Adegenet v2.0; [75]). This method first transforms the input microsatellite data into PCA loadings, then subsequently employs these uncorrelated variables as input for a discriminant analysis. The efficient summarization of high-dimensional data allows for the genetic structure among populations to be visually assessed. Sixty (of 185) PC axes explained 90.4% of the variance in the DAPC analysis. When performed on only PHAs, the first 60 (of 168) PC axes were again retained, with 92.9% of the variance explained. All discriminant axes were retained in both analyses.

### Upland game bird structure and demography

PHAs with ≥14 samples for each of three years were subsequently evaluated for genetic stability over time using assignment tests that quantified temporal structure. Demographic data for prairie chicken, to include relatedness, were previously derived by our lab [36] and employed herein. However, these data were not included in the published results for quail [37], and we thus obtained genotypes from the web (N = 434) so as to derive suitable statistics. We first calculated a population genetic baseline (diveRsify, [76]) followed by *N*_e_ and Bottleneck analyses so as to parallel the approach used for pheasant above. Recent migration rates were also calculated among assemblages using BayesAss [56].

Published analyses for relatedness in quail excluded N = 66 individuals due to the potential for consanguinity (these individuals were not identified in the original data; [77,78]). However, we deemed relatedness as an inherent component of upland game bird natural history, and hence included all individuals when we derived relatedness values (R-program related [79,80]).

## Results

### Preliminary analysis of pheasant data

Feathers were obtained from 22 PHAs (S1 Table), eight of which had insufficient sample sizes for analysis. The remaining 14 yielded 686 unique samples, with 543 successfully genotyped (*μ* = 39; Fig 2). Propagation stock also yielded 143 individuals (*μ* = 48; Table 1). Although data were generated across 24 loci, five of these were subsequently eliminated (three due to scoring issues and two others that expressed null alleles across multiple populations). Significant LD was detected for six pairs of loci, but these occurred once in four sampling groups and were non-significant following Bonferroni correction. Thus, 19 loci were employed in subsequent analyses (S2 Table).

Genetic diversity was relatively low among the three propagated stocks of pheasant, with *A*_R_ ranging from 5.9–6.6, and *H*_o_ ≤ 0.60. The stocks differed significantly among themselves with regards to *F*_ST_, and from the average *F*_ST_ for PHAs (S3 Table). Genetic diversity was also reduced within the 14 PHAs, with *A*_R_ ranging from 4.3–5.2, and *H*_O_ ≤ 0.62. Most pairwise comparisons among PHAs were significant (Bonferroni-corrected α = 0.0005), save those within the same county or immediately adjacent (S4 Table). We also found a significant pattern of IBD among PHAs (r = 0.52; P<0.002).

Additional results for pheasant population structure, demography, consanguinity, and spatial structure are reported below. This was done to facilitate comparisons with prairie chicken and quail.

### Upland game bird population structure

**Fig 3:**
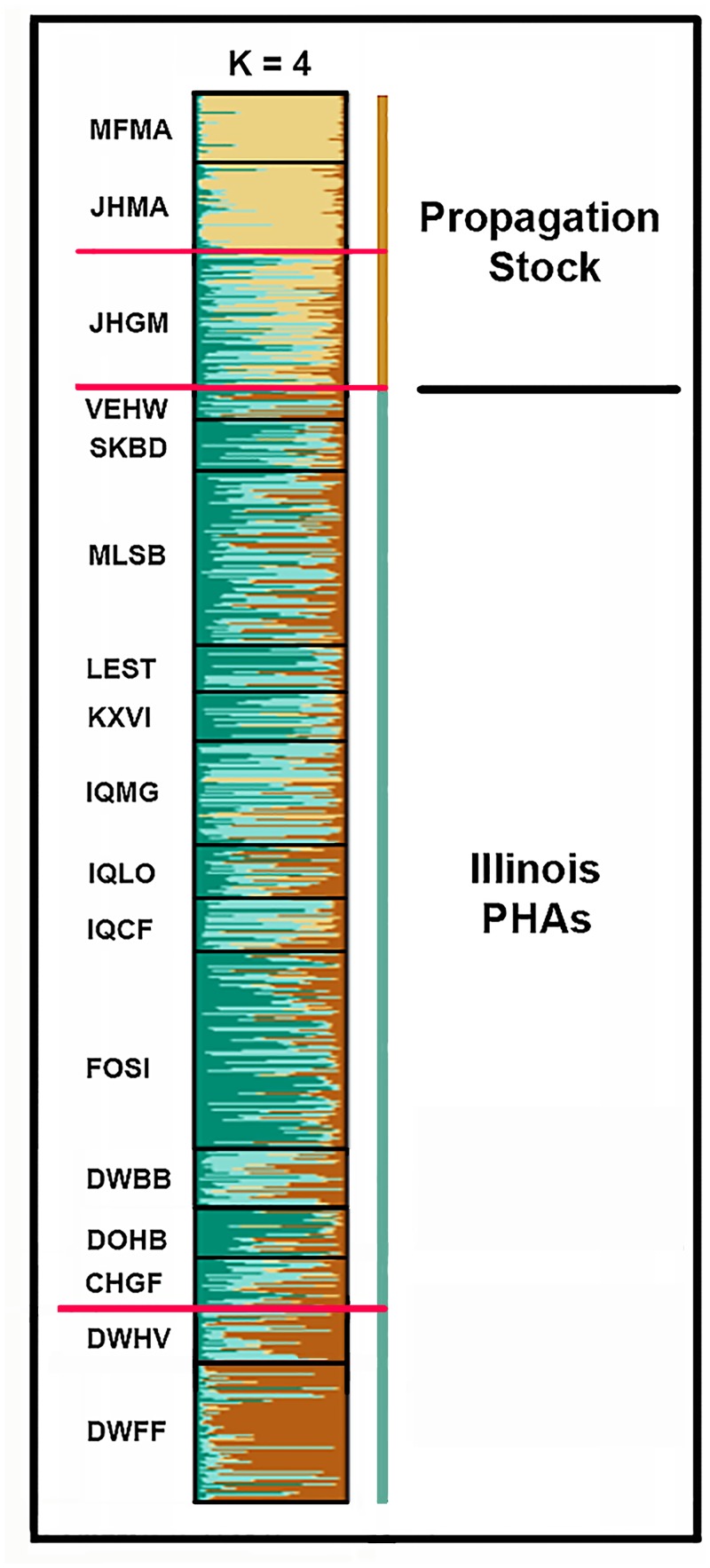
Illinois pheasant (both propagated stock and wild individuals in Illinois) assigned to group (K = 4), as determined by posterior probabilities in a Structure analysis based upon 19 microsatellite DNA loci. Lateral lines in the plot represent 686 individuals. Propagation Stock = Two groups: Manchurian (MFMA + JHMA) and Game Farm (JHGM) (Total N = 143; groups demarcated by red lines). MFMA = MacFarlane Manchurian; JHMA = J. Helfrich Manchurian; JHGM = J. Helfrich “game farm.” The remaining 14 (left column; see S1 Table for abbreviations) represent wild pheasant from Pheasant Habitat Areas (PHAs; N = 543), are partitioned into two aggregates: 12 PHAs statewide (upper) separated by a horizontal red line from two lower PHA that are isolated on all sides by interstate highways in Central Illinois; Fig 2).

For the combined propagation stock/ PHA dataset, K = 4 was selected as the best estimate for groupings, per output from the ΔK plot. Propagated stock was genetically distinct from PHAs, with the two distinct propagated stocks being Manchurian (MFMA, JHMA) and Game Farm (JHGF) (Fig 3).

Bayesian assignment tests revealed scant separation among the 14 PHAs (Fig 3), save for two that formed a distinct group in central Illinois (Fig 2). Mean migration rates between these two aggregates (*μm * =0.08%) supported their demographic isolation. Pairwise *F*_ST_-comparisons among PHAs (80/91) also indicated significant isolation (*μF*_ST_ = 0.047; Bonferroni-corrected *p*<0.0006; S4 Table). However, 10 non-significant *F*_ST_ comparisons involved a single PHA (= VEHW) that was not only significantly bottlenecked but had an extremely small sample size (N = 16; Table 1).

We then evaluated our 14 PHAs separately in Structure, without the potential influence of the three propagation stocks. Given previous results (Fig 3), we first elected to contrast ΔK and L(K) results for the PHAs, and in doing so found frequent conflicts. Given this, we subsequently treated ΔK as a “lower bound” for the number of genetic clusters, and L(K) as our “upper bound.” We also noted that K-values ≥5 rarely coincided with any discernible spatial pattern, a result subsequently corroborated by spatial structuring (below).

**Fig 4:**
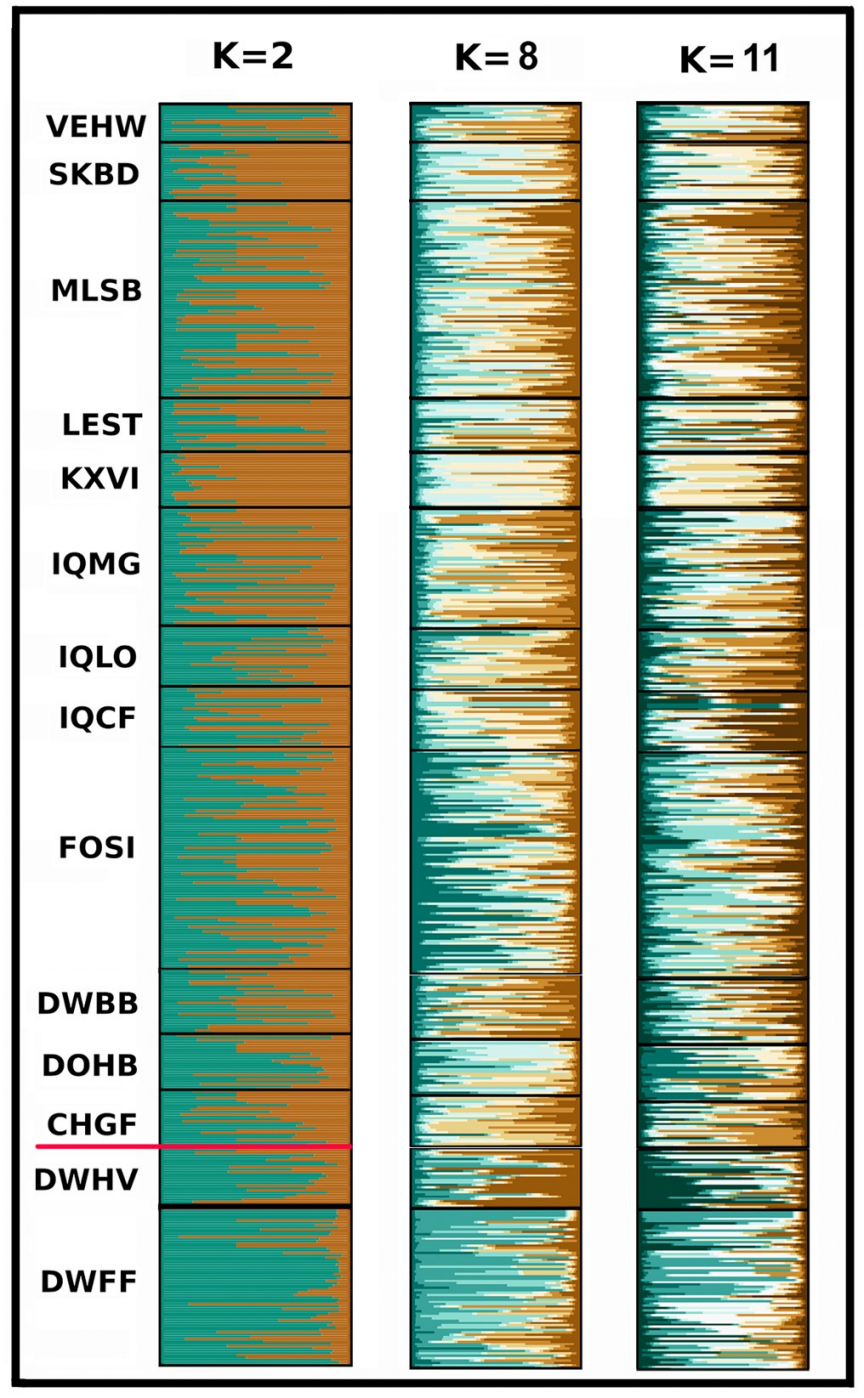
Illinois pheasant from 14 pheasant habitat areas (PHAs) assigned to group (K = 2) according to posterior probabilities in a Structure analysis based upon 19 microsatellite DNA loci. Lateral lines in the plot represent 543 individuals. Two aggregates are present, separated by a horizontal red line. The upper group contains 12 PHAs, while the lower group contains but two (DWFF and DWHV) isolated on all sides in Central Illinois by interstate highways; Fig 2).

**Fig 5:**
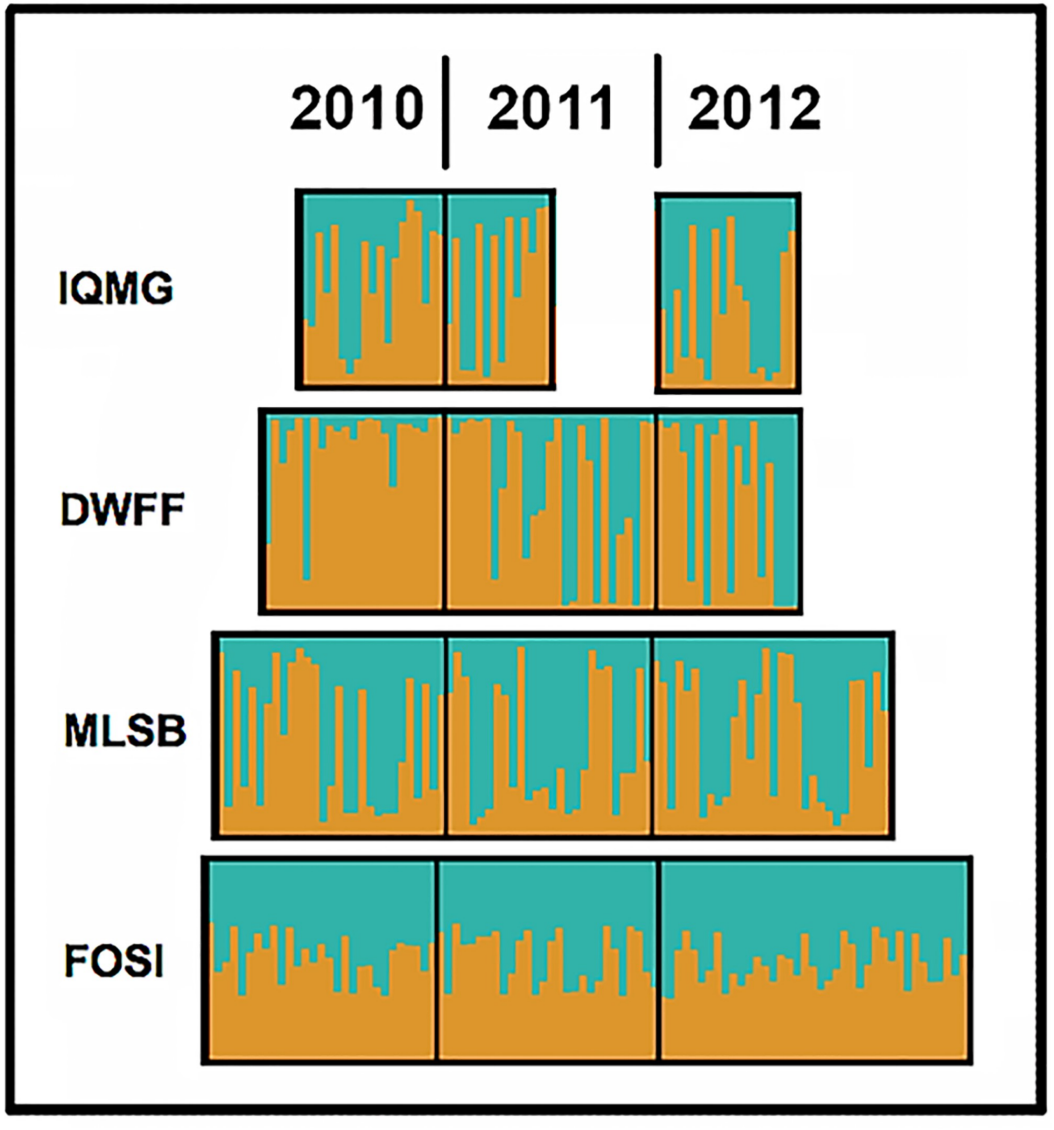
Results of a Structure analysis employing 19 microsatellite DNA loci that depict changes in genotypic frequencies across four pheasant habitat areas (PHAs) in Illinois over three consecutive years. Individuals are represented as vertical lines in plots that represent four pheasant habitat area (PHAs; left column) assigned according to year of capture (i.e., 2010/ 2011/ 2012). IQMG = Milks Grove; DWFF = Finfrock; MLSB = Saybrook; FOSI = Sibley (see S1 Table).

K = 2 emerged as the best estimate for groupings across 14 PHAs, with additional but less informative peaks recorded at K = 3, 8, and 11. The two groups were DWFF and DWHV (per Fig 3) *versus* the remaining 12 PHAs (Fig 4). We then employed Structure to evaluate the four PHAs that sustained an N≥14 over three successive years. These results (Fig 5) reflect a consistent demographic trend across years in the population structure of these PHAs.

Although prairie chicken populations differed significantly across *F*_ST_ values, leks within each were genetically similar. Likewise, quail populations also differed significantly across *F*_ST_ values [77]. We subsequently corroborated the latter result, and in so doing found but a single non-significant comparison out of 15 (Fisher’s Exact Text for sample independence with 5000 Monte Carlo replications using program diveRsify; S5 Table).

### Upland game bird demography and consanguinity

All PHAs save two were significantly bottlenecked (Table 1). Effective population size also varied among PHAs (*μ**N*_e_ = 43.3, range = 21.0–90.3; Table 1). These estimates were significantly associated with PHA area (in ha) (F_1,12_ = 10.4, *P*<0.007), thus underscoring the importance of patch size in determining upland game bird demography.

**Fig 6:**
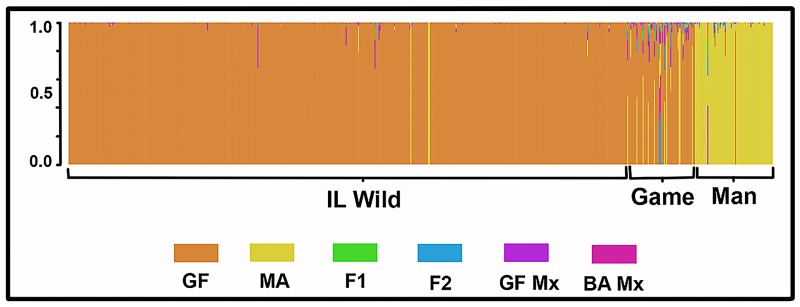
Results of a Bayesian assignment test (NewHybrids) employing 19 microsatellite DNA loci analyzed across 143 Illinois pheasant. Plot depicts potential admixture among three groups: Manchurian, game farm, and wild. Individuals are plotted as vertical lines, with potential assignment to six categories. Man = Manchurian stock derived from original brood and maintained by IL Dept. Natural Resources (IDNR); Game = Progeny of Manchurian roosters and IL Wild hens maintained by IDNR; IL Wild = Wild pheasant taken from Pheasant Habitat Areas (PHAs); GF = Game (as above); MA = Man (as above); F1 = First generation hybrid; F2 = Second generation hybrid; GF Mx = Backcross with game farm; BA Mx = Backcross with Manchurian.

Mean relatedness within PHAs (*μR* = 0.150) generally exceeded 1^st^ cousin (*R* = 0.125; Table 1), with a significant but inverse correlation between pairwise relatedness and geographic distance (*r* = -0.33; *P*<0.01). Little evidence was found for the introgression of Manchurian stock into wild populations (NewHybrids; Fig 6). Similarly, mean migration rates (*μm * =0.006%) between PHAs and propagated stock clearly supported demographic isolation.

Both prairie chicken populations had extremely low *N*_e_ values (12.7 and 13.5), with statistically significant evidence for recent and historic bottlenecks. Leks (N = 6) also reflected low *N*_e_ (*μ* = 15.9, range = 2.9–38.4), with significant bottlenecks apparent in four, and an historic signal manifested in three others. Quail populations also reflected reduced *N*_e_, comparable to that found in pheasant (*μ* = 62.1; range = 31–107). Five (of 6) were significantly bottlenecked but lacked an historic signal across generations (S6 Table).

Overall relatedness was significantly higher in quail than expected by chance alone (*p*<0.02; S1 Fig). One county (Saline) had restricted gene flow (*μm * =0.04%), and thus represented a distinct management unit [81].

### Upland game bird spatial structure

Significant IBD was also observed among PHAs (*r* = 0.52; *P*<0.002). Although population structure was predominantly global in nature (*p*<0.016), gene flow was seemingly modulated by landscape features.

Results from Geneland were somewhat reduced in that samples from PHAs and leks associated with a single UTM. Thus, genotypes could only be parsed into K-clusters by minimizing Hardy–Weinberg disequilibrium and gametic phase disequilibrium within groups. All wild pheasant were assigned to 14 clusters, representing PHAs (per Fig 3), whereas prairie chicken grouped into two populations, each representing a separate Illinois county (congruent with published results [36]). No ‘ghost’ populations or migrants were identified in either species.

**Fig 7:**
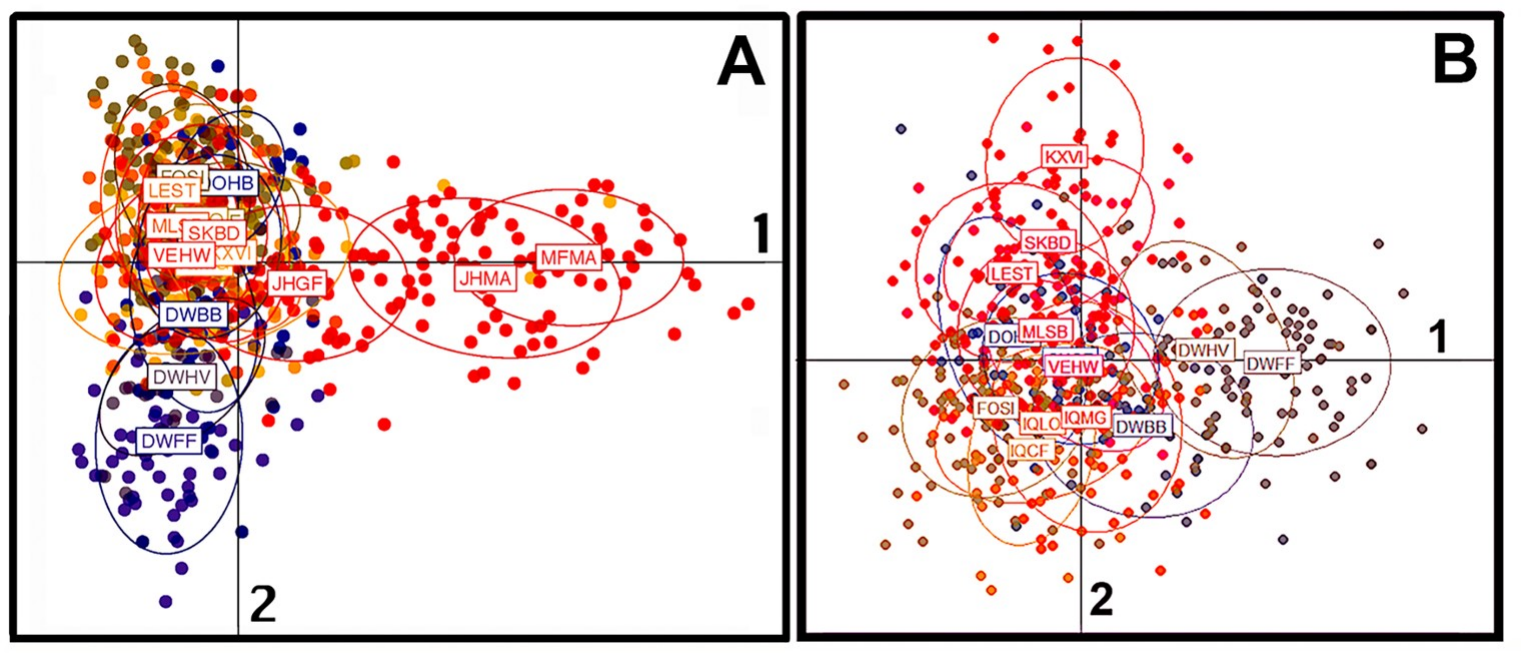
Results of a discriminant analysis of principal components (DAPC) analysis depicting differentiation among 14 pheasant habitat areas (N = 543) and three state propagated stocks (N = 143) in Illinois, as based on genotypes across 19 microsatellite loci. Part A: Two propagated stocks, i.e., MFMA (MacFarlane Manchurian) and JHMA (J. Helfrich Manchurian) are separated on discriminant axis 1 from the third propagated stock (JHGF; J. Helfrich “game farm”) as well as the 14 PHAs. Axis 2 distinguishes DWFF (Finrock PHA; S1 Table) from the remainder. Part B: Two PHAs, i.e., DWFF and DWHV (Hallsdale PHA; S1 Table) separate on axis 1 while KXVI (Victoria PHA; S1 Table) is not consideration distinct at the apex of axis 2 as it is scattered quite diffusely.

Our DAPC analyses included both PHAs and captive broodstock, with 60 (of 185) PC axes retained, explaining 90.4% of the variance in the data. A plot of discriminant axes 1 and 2 (Fig 7A) depicts both propagated Manchurian stocks (i.e., MFMA and JHMA) as relatively distinct on axis 1 and separated from the third propagated stock (i.e., JHGF), as well as the 14 PHAs. The second axis separates DWFF (Finrock PHA) from the remaining 13 PHAs.

The results of a similar analysis, performed only with the 14 PHAs, is presented in Fig 7B. The first 60 (of 168) PC axes were again retained, explaining 92.9% of the variance. DWFF is again relatively discrete on axis 1, with DWHV (Hallsdale PHA) somewhat peripheral on that axis as well. KXVI (Victoria PHA) and DWBB PHA (Birkbeck; S1 Table), at top and bottom of axis 2, respectively, are diffusely scattered with weak separation.

### Upland game bird comparisons

Pheasant, quail, and state-endangered prairie chicken differed with regard to numbers of aggregates and individuals (Table 2). Pairwise aggregations within each species also differed significantly. Pheasant and prairie chicken reflected significant IBD whereas quail did not. Structure analyses clearly separated prairie chicken populations, but not quail or pheasant, yet aggregates within each differed significantly when compared using *F*_ST_-tests. Average heterozygosity, suggestive of short-term survival, did not differ significantly among the three, whereas allelic diversity did (suggesting a diminished long-term survival). In summary, population genetic parameters clearly juxtapose across all three species, and this uniformity is driven largely by chronic anthropogenic disturbance.

**Table 2:** Comparison of population genetic and life-history parameters gauged among populations of wild pheasant (pheasant habitat areas = IL PHAs), greater prairie chicken (= IL GRPC), and bobwhite quail (= IL BWQ) in Illinois.

Parameter	IL PHAs	IL GRPC	IL BWQ
Msats assayed	19	21	11
*F*_ST_ Differentiation	100%	100%	100%
% Bottlenecked	86%	100%	86%
Average *N*e	43	13	62.1
Average R	0.15	0.31	0.02
Average *H*o	0.62	0.65	0.71
Average *A*r	5.34	5.35	8.63
Breeding structure	Polygyny	Polygyny	Monogamy
Clutch size	7–15	5–17	7–28
Brood number	1–2	1	1–3
Incubation days	23–28	23–25	22–24
Adult size	60–89 cm	43 cm	24–28 cm
Adult weight	0.9–1.2 kg	0.7–1.2 kg	0.13–0.17 kg

Msats = microsatellite DNA markers; *F*_ST_ Differentiation = significant *F*_ST_ values; *N*e = Effective population size; R = Relatedness; *H*o = Observed heterozygosity; *A*r = Allelic richness.

## Discussion

### Management priorities

Biodiversity must be managed cooperatively so as to minimize costs and optimize investments, particularly when evaluating anthropogenically-modified regions such as Midwestern North America. This framework often provides the basis for an MTM plan, pending additional covariates such as landscapes, phylogenetic relationships, dispersal capacities, and economic appraisals [13,82,83]. Yet, when this is done, an overly complex plan often emerges such that effectiveness becomes an overriding concern. For example, can a few covariates effectively parse numerous species? Is it effective when compared with alternatives? [84]. Our approach to this issue involved the development of an MTM that included a series of precise and consistent metrics as its basis, and we summarize our results below.

### Context-specific options

#### Propagation stocks

One concern with captive propagation is that genetic and demographic repercussions quickly surface when stocks or introductions are inappropriately managed [85]. For example, deleterious and partially recessive alleles are often sustained within brood stock due to the relaxed selection inherent to propagation facilities [86]. As an example, the propagated pheasant stock in this study were found to be significantly bottlenecked.

Our results also underscored significant relatedness among individuals, another compounding issue for propagation stock. The original Manchurian broodstock (MFMA) were most closely related (at half-sib, *R* = 0.24), whereas Illinois Manchurian (JHMA) exceeded first cousin (*R* = 0.143). These metrics are particularly relevant in that captive-reared parents are often bred iteratively for supplementation purposes, a practice that not only diminishes the effectiveness of propagated stock, but more importantly, impacts the fitness of progeny.

A potential remediation would be to constrain the time in captivity for parental stock [87]. Alternatively, controlled hunts could also be managed more sustainably by relying on wild rather than propagated individuals as a means of supplementation [88], an approach successfully employed in fisheries management. However, one important question for such a strategy is whether wild populations can adequately sustain the loss of adults so as to bolster reintroductions into controlled hunt areas.

#### Hunter-harvest

Several questions emerge when the genetic consequences of exploitative hunting are discussed [31]. Can it be detected and mitigated? Does it indeed impact demography and yield? A response to the first question [31] was primarily circumstantial, whereas the second response was ‘under consideration.’

Hunter-harvest (particularly with a focus on body size and/or gender) can have serious demographic impacts, and these are consistently reflected in population genetic parameters. For example, a modeling exercise on the dynamics of a well-researched Fennoscandian moose population [89] demonstrated that the selective harvest of males promoted genetic drift in each subsequent generation, concomitant with a reduction in *N*_e_. These effects persisted despite a consistent population growth, and without considering the potential for individual differences in male quality (i.e., all males treated equally).

In this study, we attempted to quantify ‘detection’ and ‘impact’ by utilizing a population genetic framework in our evaluation of wild and stocked pheasant. We also addressed the question of ‘mitigation’ by applying (and subsequently comparing) our metrics across additional upland game bird species (i.e., prairie chicken and quail).

### Population genetics of upland game birds

We found that PHAs were not only isolated from one another, but with fluctuating demographics as well, as evidenced by significant differences in *F*_ST_, relatedness, and bottleneck values. There was no gene flow from propagated stock to PHEs, despite the fact that CHRs (Fig 2) were annually supplemented with thousands of captive-bred individuals. This suggests two possibilities: hunting pressure is substantial in the CHRs, with population densities relatively depressed as a result. This would potentially reduce the competitive pressure that may result from substantial and iterative restocking. Alternatively, hunting pressure is reduced, but few individuals survive the winter season. Both scenarios would sustain the limited emigration observed from CHRs to PHAs.

Relevant population genetic parameters for T&E species such as prairie chicken are often difficult to derive, due largely to the inherent difficulties with sampling. However, we managed to derive genotypes at 19 microsatellite loci by extracting DNA from feathers shed on leks [36]. Our results pointed to significant demographic isolation, bottlenecks, minimal migration rates (*m*<1%), with *N*_e_ values quite low (4-year *μ* = 13.1). Relatedness and inbreeding values were also significantly elevated, with dispersal constrained by the presence of dominance hierarchies within leks, as manifested by 12 significantly different family groups (*R* = 0.31). Genetic patterns in prairie chicken clearly parallel those found in pheasant and do so despite potential life-history differences (Table 2).

Quail, a third management target, is hunter-harvested as well as being propagated within state facilities (yet the latter were not assayed [37]). Instead, wild, hunter-harvested individuals (N = 434) were sampled across six Illinois counties. Results parallel those found in pheasant and prairie chicken, with populations significantly isolated (S5 Table), bottlenecked (S6 Table), and more closely related than by chance alone (S1 Fig). Gene flow and population structure were also alarmingly depressed in quail [77,78], with IBD being apparent. However, interstate highways seemingly had little discriminatory effect on quail, mirroring a similar result in pheasant where 86% of PHAs were unaffected (save two surrounded on all sides by interstates).

Quail is also sedentary and ground-dwelling, with a strong communal instinct and a low capacity for dispersal [39]. An effective management plan for this species would be to acquire additional habitat adjacent to currently occupied sites (see sharing/sparing below). This approach would also juxtapose well with quail life history by providing additional habitat proximal to family groups.

These results, in combination, provide a clear understanding of the manner by which chronic anthropogenic disturbance negatively impacts population structure, demography, and landscape genetics of upland game birds in midwestern North America. As such, they represent a major challenge for wildlife management, particularly given the ongoing mandate that a given conservation investment should indeed elicit an optimized economic return [90].

### Can a genetically informed MTM be effective?

Systems that incorporate multiple threats and species (as herein) are difficult to prioritize and monitor, and this in turn impacts an effective return from conservation expenditures. To be successful in this regard, an MTM must not only be robust (per population genetic metrics), but also flexible enough to sustain future activities and decisions. One mechanism is to employ the Open Standards for the Practice of Conservation [91], with goals that would modify/increase prairie habitat patches, establish their inter-connectivity, and promote ‘no-take’ reserves that prevent hunter-harvest. Here, the conservation ‘targets’ would be prairie-obligate birds, with measurable ‘indicators’ being precise and consistent population genetic metrics. Below, we outline the manner by which these goals can be attained for upland game birds, particularly within the extensive agroecosystem of midwestern North America.

Clearly, the resource most limiting in this situation is habitat. Agriculture occupies 40% of global ice-free land [92], and is regarded as the single greatest threat to global biodiversity [93]. Impacts on biodiversity will be extensive, given that anthropogenic food demands will double in a scant few decades [14]. Consequently, it is imperative that methodologies be employed within the context of Open Standards that will actively and collaboratively integrate both agroecosystems and biodiversity. One such solution is the concept of ‘land sharing,’ where both components coexist within the same landscape. A second is ‘land sparing,’ a process that effectively isolates biodiversity from an agricultural matrix [94].

#### Land-sharing

Here, emphases are two-fold: to retain small patches of unfarmed natural or semi-natural vegetation within larger agricultural plots, and to reduce the negative effects of mechanized agriculture on lands adjacent to these plots [95]. The approach is particularly germane for inherently large midwestern agroecosystems where monoculture predominates. Less productive agricultural areas are not only available in this context, but also sufficient to accommodate native vegetation as well as edge habitat that allows for dispersal and connectivity (per Open Standards). Downsides include a potential reduction in agricultural yields, as well as the habitat degradation that can emerge when small, segregated plots are gradually engulfed by an expanding agroecosystem [21].

Land-sharing was once prevalent in midwestern North America, with small circa-1950 farms producing grains, hay, and livestock within fields demarcated by fencerows. But a generational shift in agricultural efficiency has resulted in these smaller, more marginal plots of habitat being lost. Now, crops are predominantly corn/soybean interspersed by pastures/waterways of dense, cool-season brome/fescue unfavorable for upland game birds. Agricultural efficiency has promoted the incorporation of topographically more diverse habitat as well. Yet, these situations can be easily rectified. The less-productive edge-habitat, largely subsumed by more efficient agricultural methods, can be easily re-established [41], and with but minor reductions in agricultural yield as a result.

We recognize that edge habitats will not sustain large, genetically diverse populations of upland game birds, but they will provide the corridors necessary for dispersal among currently isolated fragments. Elevated connectivity and its resulting gene flow would not only counteract demographic isolation but at the same time leverage those genetic metrics already depressed, such as migration rates, bottleneck effects, and reduced *N*_e_. Those negative rates that currently characterize our three study species [i.e., elevated relatedness (*R*) and inbreeding (*F*)] would also be reversed as well.

#### Land-sparing

Rather than modify existing agroecosystems for the benefit of wildlife (as above), the land-sparing approach emphasizes the protection/ restoration of as much native vegetation as possible. Establishing sufficiently large ‘no-take’ reserves, for example, would foster larger population sizes of upland game birds, provide the necessary refugia, as well as provide a buffer against ongoing climate change [96]. However, success is contingent upon two factors: individual movements, as well as reserve size, as both promote genetic diversity [97]. In this study, elevated *N*_e_ values for pheasant were significantly associated with larger PHAs (μ*N*_E_ = 43; P<0.007). A potential downside would be if agriculture was intensified on neighboring plots. This, in turn, would entail greater use of agrochemical, water, and energy resources [98]. Production costs become elevated while the quality of habitat in adjacent plots is simultaneously reduced.

Land-sparing is a positive concept for biodiversity, but only if land is actually ‘spared.’ Two limitations are apparent: spared land is not actually utilized for conservation (i.e., incomplete area sparing), and/or its quality may have diminished following an earlier assessment (i.e., lower habitat quality sparing). Despite these limitations, land-sparing still outperforms land-sharing, but only as long as ≥28% of the land is devoted to conservation, and if ≥29% of its original quality is retained [99].

For upland game birds, land sparing would allow additional populations to be established, an aspect particularly important for prairie chicken in that only two remain in Illinois. It would also promote larger, demographically more stable populations in all three study species. This, in turn, would enhance population genetic metrics such as *F*_ST_ and *N*_e_, while also buffering against bottlenecks.

#### The Conservation Reserve Program (CRP)

Land sparing actually has a recognized legacy in the Midwest. The primary goal of the Conservation Reserve Program (CRP), a provision of the 1985 U.S. Farm Bill, was to ensure food security in the United States. In doing so, it provided a 10-year subsidy for removal of crops from farmland deemed suboptimal [100], and given this, wildlife habitat and water quality were enhanced, but only as an indirect effect.

A point of contention is that CRP acreage fluctuates in response to market conditions, with expansion occurring as agricultural prices drop, and retraction as they rise. For example, CRP land declined 35% from 2007 to 2014 (i.e., from14.9 to 9.7 million ha) [101]. These impacts were compounded as well: not only did we lose previously conserved land, but the increase in cultivation served to exacerbate global carbon emissions and depress ecosystem services [102]. An additional negative is that honeybee forage was similarly reduced, a situation concomitant with a reduction in colony numbers [103].

Despite early (and indirect) success, the CRP is now in a steady decline as agricultural prices escalate [104]. CRP-plots become strongly transitional as a result, (i.e., available only for a limited time), and thus are of limited conservation value. The situation may actually be detrimental, in that bottlenecks in resident species are iteratively induced as habitat is consistently reduced. This allows the same negative demographic mandates to again emerge: i.e., depressed *N*_e_, heightened inbreeding, and elevated relatedness.

In summary, the CRP program provides upland birds with only indirect and intermittent benefits, due largely to the variance in (and loss of) allocated land. An additional discrepancy is the unregulated management of plots once so incorporated. In this sense, inappropriate plantings frequently occur, and non-native (pioneer) vegetation is allowed to encroach. In addition, the frequent mowing that occurs as a control mechanism for non-natives also serves to block natural succession.

The CRP clearly has an uncertain future, particularly given elevated commodity prices and ongoing energy developments that work in tandem to reduce its scope (available from: https://www.dnr.illinois.gov/publications/documents/00000716.pdf). However, a land sharing/sparing program, one that incorporates a rigorous framework coupled with a long-term management plan, would not only sustain current CRP parcels but also sustain on a long-term basis those indirect conservation benefits that are now in serious decline.

### Will land-sparing benefit upland game birds?

#### Common pheasant

Direct benefits of land-sharing have yet to be recorded for upland game birds, although they can be extrapolated from those that have been gleaned from CRP-lands. In this sense, pheasant has directly benefitted from the high-diversity seed mix that was often employed on CRP-managed parcels, not only as a trophic component, but also as a means to promote pasture and small grain habitat [105,106,107,108]. However, these CRP-derived commodities are diminishing due to ongoing agroecosystem expansion. This not only reduces midwestern grasslands [109], but secondarily impacts pheasant. Despite the bleak prognosis, previous results clearly demonstrate that a well-thought out land-sharing portfolio would directly benefit pheasant in particular, and grasslands in general.

#### Northern bob-white quail

Quail, on the other hand, clearly associates with farmland habitat, and its abundance is demonstrably promoted by the greater proportion of herbaceous vegetation found on CRP-land [110,111]. This underscores an important point: a necessary requirement for a land-sparing portfolio, particularly in the Midwest and Southeast, is the active promotion of early successional native plant communities [112]. Positive results for quail are achieved when management is proactive, and this means the elimination of dense exotic grasses while simultaneously optimizing edge habitat and open spaces [113]. Quail have repeatedly responded in a positive manner to well-managed CRP-parcels and will continue to do so if a well thought out land-sparing portfolio is employed.

#### Greater prairie chicken

Breeding leks of prairie chicken have also increased as a direct result of the land-use characteristics most often promoted by CRP-lands (i.e., smaller residential-farmstead plots, reduced forest patches, and more expansive habitat parcels) [114]. Promoting an increase in the number of leks will also elevate the numbers of resident and competing males [115], an important consideration given the presence of dominant family groups [36]. Similarly, the retention of abundant grass and forb cover on CRP-fields also serves to promote nest survival in prairie chicken [116], a situation that similarly resonates with pheasant and quail. In summary, CRP-protected lands are a positive asset for prairie chicken, particularly when they are adjacent to grasslands, and when invasive plants are actively suppressed [113]. However, these requisites must become a management baseline within the land-sparing portfolio of an upland game bird MTM.

#### Edge habitat across species

A proactive focus on edge habitat as a component of land-sharing will provide ecosystem services comparable to those found in more standard reference systems [117]. In addition, small patch sizes inherent to land-sharing can easily sustain small-scale generalists such as quail, with limited dispersal ability but a broader tolerance for agricultural practices [21]. These aspects can be enhanced when edges are placed adjacent to and/ or connected with a land-sparing component, such as that currently employed with Illinois PHAs. They also blunt small population effects, such as bottlenecks, low *N*_e_, and elevated relatedness, each of which is currently manifested across our study species. The coupling of edge-habitat with a land-sharing augmentation would also stimulate upland game bird demographics by optimizing offspring survival, genetic variability, and *N*_e_, while minimizing variance in reproductive success.

### A potential solution

Conservation decisions are driven by economics and public concern, and these reverberate equally among policy makers and stakeholders. For sure, resources become limited and conflicts subsequently emerge when management objectives and societal needs overlap (such as with agricultural yields and recreational hunting). An economic baseline must clearly reside within an MTM, and although positive manifestations are recognized (as above), their delivery may be more difficult. Land-sparing would be less pressing of an issue if a land-sharing component could become a more viable approach. Instead, land-sparing is deemed the best approach to accommodate both agricultural production and biodiversity conservation, particularly for species with more restricted global distributions [98].

Thus, an MTM plan for upland game birds, one that would promote biodiversity and alleviate habitat loss/modification, must by necessity incorporate a land-sparing strategy. Similarly, it would also be divorced by necessity from the declining and market-driven CRP, where management directives are weakly established with regard to allocated land, whereas linkages with agricultural commodity pricing are quite strong.

On the positive side, the partitioning of land to protect biodiversity is recognized as a new global initiative [for example, The Half-Earth Concept [118] and Nature Needs Half (available from: https://natureneedshalf.org/nature-needs-half/)]. Both require greater yields from areas already under cultivation, a process that may best be implemented at the state-level. In addition, funds now allocated for single-species conservation, and for propagation facilities that sustain a put-and-take hunter-harvest [119], could potentially be pooled as a cost-saving to initiate a state or region-wide MTM. This would also offset potential losses that may stem from a strong land-sparing mandate. The objective is contemporary management at the wildlife-agroecosystem interface, but importantly, with grasslands conserved, upland game birds promoted, and agricultural production sustained without the loss of additional habitat.

## Supporting information

S1 Fig Relatedness values (Wang, 2002) derived for 434 hunter-harvested northern bobwhite quail distributed across six aggregates in southern Illinois. (TIFF)

S1 Table Location (latitude/ longitude) and size (in ha) for 22 Illinois pheasant habitat areas (PHAs). (PDF)

S2 Table Description of microsatellite (msat) DNA primers used in the study. This includes number of sampled genotypes, alleles detected, multiplex assignment, annealing temperature, and violations of Hardy-Weinberg equilibrium (HWE) for each locus.
(PDF)

S3 Table Pairwise *F*_ST_ values calculated for common pheasant propagation stocks and wild populations (ILWI) in Illinois. (PDF)

S4 Table Pairwise *F*_ST_ values calculated for common pheasant sampled from 14 Illinois pheasant habitat areas (PHAs). (PDF)

S5 Table Fishe’s Exact tests for pairwise independence of hunter-harvested northern bobwhite quail aggregations in Illinois. (PDF)

S6 Table Bottleneck and effective population size (Ne) estimates recorded for hunter-harvested northern bobwhite aggregations in Illinois. (PDF)
