# Supplementary figures and images for: Multi-targeted management of upland game birds at the agroecosystem interface in midwestern North America
Source: PLoS One. 2020 Apr 27;15(4):e0230735. doi: 10.1371/journal.pone.0230735 (PMC7185590; doi:10.1371/journal.pone.0230735)

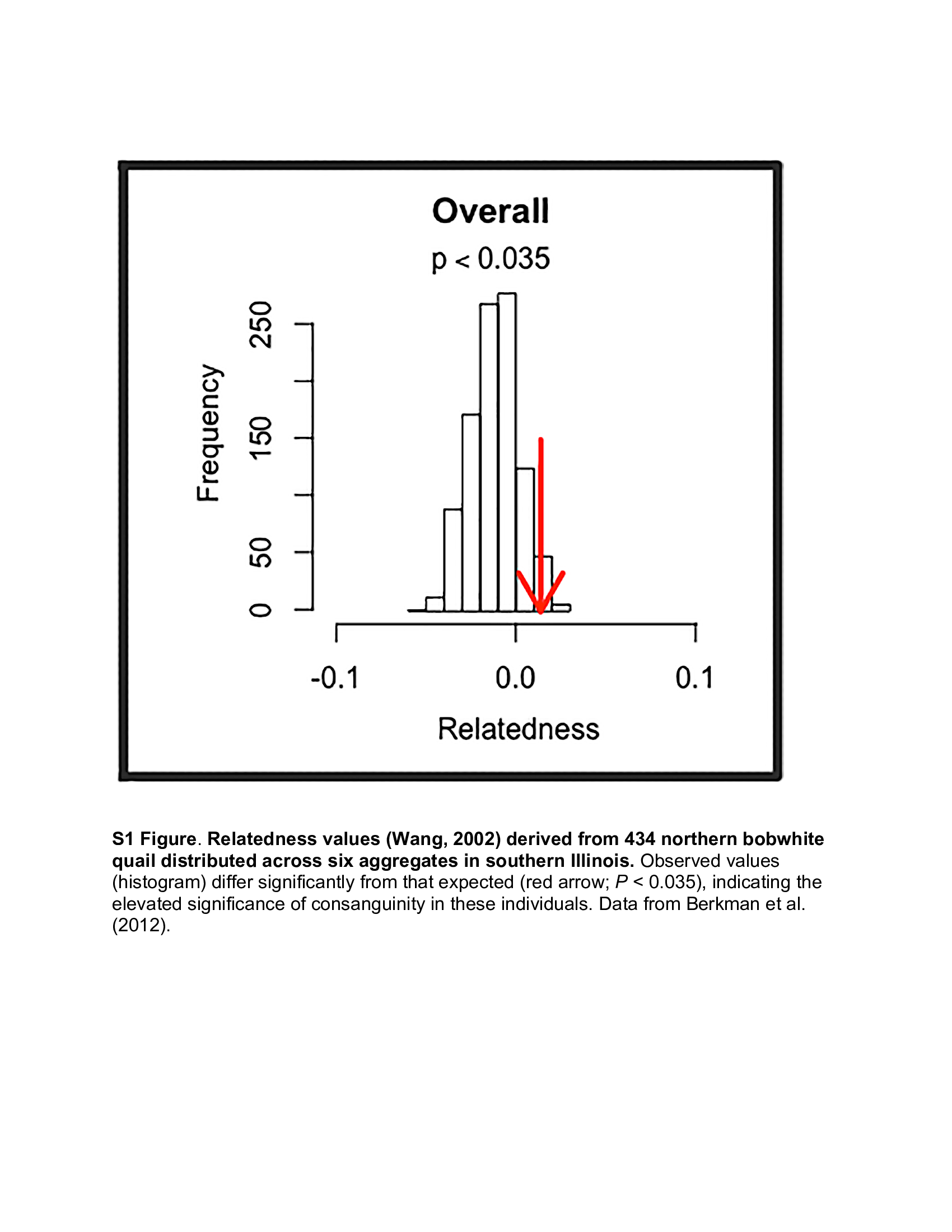

Supplement: S1 Fig — (TIFF) [file pone.0230735.s001.tiff]
