# Supplementary material for: Multi-targeted management of upland game birds at the agroecosystem interface in midwestern North America
Source: PLoS One. 2020 Apr 27;15(4):e0230735. doi: 10.1371/journal.pone.0230735 (PMC7185590; doi:10.1371/journal.pone.0230735)
Supplement: S1 Table — (PDF) [file pone.0230735.s002.pdf]

Table S1. Location (latitude/ longitude) and size (in ha) for 22 Illinois Pheasant Habitat Areas (PHAs). Those with asterisks (*) were not included in the present study due to small sample sizes. PHA = sampling location; Code = PHA abbreviation; Area = size of PHA in ha (mean below column). Geographic location of PHAs is depicted in Figure 1.

PHA	Code	County	Latitude	Longitude	Area (ha)
Gifford	CHGF	Champaign	40.323586	88.055097	40.7
Hindsboro	DOHB	Douglas	39.688629	88.072402	35.6
Birkbeck	DWBB	DeWitt	40.203603	88.869284	32.4
Finfrock	DWFF	DeWitt	40.256927	89.098724	147.7
Hallsville	DWHV	DeWitt	40.162853	89.097951	33.6
Perdueville*	FOPV	Ford	40.405214	88.206385	48.6
Sibley	FOSI	Ford	40.566235	88.394514	255
Clifton	IQCF	Iroquois	40.940914	-87.89757	32
Loda	IQLO	Iroquois	40.528284	88.046168	64.8
Milks Grove	IQMG	Iroquois	40.928118	88.039939	30.8
Victoria	KXVI	Knox	41.047332	90.116951	108
Sand Prairie*	LESP	Lee	41.632120	89.622332	127.9
Steward	LEST	Lee	41.820560	88.975681	32.4
Whitefield*	MAWF	Marshall	41.109028	89.500636	47.8
Freeman Mine*	MGFM	Montgomery	39.437939	89.633609	72.4
Saybrook	MLSB	McLean	40.463168	88.552677	261.4
Oakford*	MNOF	Menard	40.928118	88.039939	40.5
Sand Ridge*	MSSR	Mason	40.390756	89.870822	121.4
Dublin Highlands*	SHDH	Stephenson	42.291829	-89.77258	40.4
Bradford	SKBD	Stark	41.157816	89.737085	41.7
Ilo Dillin*	TAID	Tazewell	40.374932	89.341016	32.4
Herschel Workman	VEHW	Vermillion	40.459376	87.920044	57.1
					77.48
