# Supplementary material for: Multi-targeted management of upland game birds at the agroecosystem interface in midwestern North America
Source: PLoS One. 2020 Apr 27;15(4):e0230735. doi: 10.1371/journal.pone.0230735 (PMC7185590; doi:10.1371/journal.pone.0230735)
Supplement: S2 Table — This includes number of sampled genotypes, alleles detected, multiplex assignment, annealing temperature, and violations of Hardy-Weinberg equilibrium (HWE) for each locus. (PDF) [file pone.0230735.s003.pdf]

S2 Table. Microsatellite DNA primers used to successfully amplify common pheasant feather samples. Listed are Locus, number of samples genotypes (N), number of alleles detected (#A), Reference for each primer, multiplex panel assignment (MPx), annealing temperature (T_A), and number of sampling groups in violation of Hardy-Weinberg equilibrium (HWE) for each locus.

Locus	N	#A	Reference	MPx	T_A	HWE
MCW55	1306	4	Baratti et al. (2001)	1	55	1
SR11*	1263	19	Cheng et al. (1995)	1	55	-
TUT02	1304	16	Segelbacher et al. (2000)	1	55	0
ADL230*	1302	11	Wang et al. (2009)	1	55	-
MNT408	1306	15	Bech et al. (2010)	2	59	0
MNT477	1306	5	Bech et al. (2010)	2	59	0
Aru1A1	1192	10	Ferrero et al. (2007)	2	59	0
SR01	1306	10	Wang et al. (2009)	2	59	0
ADL292	1306	4	Bech et al. (2010)	3	55	0
MNT467	1306	4	Bech et al. (2010)	3	55	0
SR03	1297	11	Wang et al. (2009)	3	55	3
SR19	1306	11	Wang et al. (2009)	3	55	0
1H4	673	11	Gu et al. (2012)	8	52	4
3D2	681	9	Gu et al. (2012)	8	52	3
4H1	685	3	Gu et al. (2012)	8	52	0
5H7	686	15	Gu et al. (2012)	8	52	0
TT06	683	4	Gu et al. (2012)	8	52	2
2420	684	11	Gu et al. (2012)	9	55	0
2580	681	18	Gu et al. (2012)	9	55	0
4C12	686	8	Gu et al. (2012)	9	55	1
4F8	681	22	Gu et al. (2012)	9	55	3
