# Supplementary material for: Multi-targeted management of upland game birds at the agroecosystem interface in midwestern North America
Source: PLoS One. 2020 Apr 27;15(4):e0230735. doi: 10.1371/journal.pone.0230735 (PMC7185590; doi:10.1371/journal.pone.0230735)
Supplement: S3 Table — (PDF) [file pone.0230735.s004.pdf]

S3 Table. Pairwise F_{ST} values calculated for common pheasant in Illinois. Samples represent wild individuals (N=686) across 14 Pheasant Habitat Areas (PHAs), plus individuals (N=543) representing three propagation stocks [J. Helfrich Manchurian (JHMA), J. Helfrich "game farm" (JHGF), and MacFarlane Manchurian (MFMA); N=143]. Estimates were derived from microsatellite DNA marker panels composed of 19 loci. F_{ST} values are below the diagonal, while *P*-values are above. All tabulated F_{ST} values are significantly different (*) following Bonferroni correction (α = 0.008). Samples sizes are in Table S1.

	PHA	JHGF	JHMA	MFMA
PHA	-	≤0.0001	≤0.0001	≤0.0001
JHGF	0.025*	-	≤0.0001	≤0.0001
JHMA	0.084*	0.045*	-	≤0.0001
MFMA	0.126*	0.084*	0.032*	-
