# Supplementary material for: Multi-targeted management of upland game birds at the agroecosystem interface in midwestern North America
Source: PLoS One. 2020 Apr 27;15(4):e0230735. doi: 10.1371/journal.pone.0230735 (PMC7185590; doi:10.1371/journal.pone.0230735)
Supplement: S4 Table — (PDF) [file pone.0230735.s005.pdf]

S4 Table. Pairwise F_{ST} values calculated for 543 common pheasant sampled from 14 Illinois Pheasant Habitat Areas (PHAs). The F_{ST} values are presented below the diagonal, while *P*-values are above the diagonal. The F_{ST} values denoted by an * represent evidence of significant divergence after Bonferroni correction (α = 0.0006). Estimates are derived from 19 microsatellite DNA loci. Samples sizes and locality information are provided in Table 3.

	CHGF	DOHB	DWBB	DWFF	DWHV	FOSI	IQCF	IQLO	IQMG	KXVI	LEST	MLSB	SKBD	VEHW
CHGF	-	≤0.0001	≤0.0001	≤0.0001	≤0.0001	≤0.0001	≤0.0001	0.0041	≤0.0001	≤0.0001	≤0.0001	≤0.0001	≤0.0001	0.0334
DOHB	0.037*	-	≤0.0001	≤0.0001	≤0.0001	≤0.0001	≤0.0001	≤0.0001	≤0.0001	≤0.0001	≤0.0001	≤0.0001	≤0.0001	0.0004
DWBB	0.038*	0.062*	-	≤0.0001	≤0.0001	≤0.0001	≤0.0001	≤0.0001	≤0.0001	≤0.0001	≤0.0001	≤0.0001	≤0.0001	0.0049
DWFF	0.062*	0.066*	0.044*	-	≤0.0001	≤0.0001	≤0.0001	≤0.0001	≤0.0001	≤0.0001	≤0.0001	≤0.0001	≤0.0001	≤0.0001
DWHV	0.066*	0.097*	0.057*	0.053*	-	≤0.0001	≤0.0001	≤0.0001	≤0.0001	≤0.0001	≤0.0001	≤0.0001	≤0.0001	≤0.0001
FOSI	0.043*	0.039*	0.042*	0.060*	0.072*	-	≤0.0001	≤0.0001	≤0.0001	≤0.0001	≤0.0001	≤0.0001	≤0.0001	0.0007
IQCF	0.045*	0.061*	0.040*	0.052*	0.065*	0.031*	-	≤0.0001	≤0.0001	≤0.0001	≤0.0001	≤0.0001	≤0.0001	0.0026
IQLO	0.016	0.035*	0.030*	0.051*	0.066*	0.034*	0.027*	-	≤0.0001	≤0.0001	≤0.0001	≤0.0001	≤0.0001	0.0607
IQMG	0.037*	0.050*	0.027*	0.045*	0.057*	0.026*	0.026*	0.031*	-	≤0.0001	≤0.0001	≤0.0001	≤0.0001	0.0246
KXVI	0.069*	0.062*	0.068*	0.077*	0.096*	0.046*	0.064*	0.068*	0.059*	-	≤0.0001	≤0.0001	0.00031	≤0.0001
LEST	0.049*	0.054*	0.059*	0.081*	0.102*	0.045*	0.077*	0.065*	0.056*	0.056*	-	≤0.0001	≤0.0001	0.002
MLSB	0.029*	0.043*	0.028*	0.043*	0.053*	0.028*	0.027*	0.022*	0.028*	0.039*	0.038*	-	≤0.0001	0.019
SKBD	0.047*	0.043*	0.049*	0.069*	0.097*	0.046*	0.057*	0.050*	0.043*	0.027	0.037*	0.034*	-	≤0.0001
VEHW	0.013	0.031	0.019	0.039*	0.055*	0.018	0.023	0.01	0.013	0.048	0.032	0.011	0.032*	-
