# Supplementary material for: Multi-targeted management of upland game birds at the agroecosystem interface in midwestern North America
Source: PLoS One. 2020 Apr 27;15(4):e0230735. doi: 10.1371/journal.pone.0230735 (PMC7185590; doi:10.1371/journal.pone.0230735)
Supplement: S5 Table — (PDF) [file pone.0230735.s006.pdf]

S5 Table. **Fisher's Exact tests for pairwise independence of northern bobwhite quail aggregations in Illinois.** Data represent genotype counts derived from 11 microsatellite (msat) DNA loci across 434 individuals. Monte Carlo replications = 5000; Counties = Marion (MAR), Saline (SAL), Washington (WSH), Wayne (WAY), Scott (SCO), Perry (PER); N = All pairwise comparisons were significant save for that indicated in bold text and with an asterisk. Data from Berkman et al (2012).

Counties	N	MAR	SAL	WSH	WAY	SCO	PER
MAR	122	X					
SAL	137	0	X				
WSH	78	0	0	X			
WAY	52	0.0188	0	0	X		
SCO	23	**0.1005***	0.049	0.0046	0.0088	X	
PER	22	0.005	0.0002	0.0034	0.0001	0.0196	X
