# Supplementary material for: Multi-targeted management of upland game birds at the agroecosystem interface in midwestern North America
Source: PLoS One. 2020 Apr 27;15(4):e0230735. doi: 10.1371/journal.pone.0230735 (PMC7185590; doi:10.1371/journal.pone.0230735)
Supplement: S6 Table — (PDF) [file pone.0230735.s007.pdf]

**Table S6**. **Results of a two-tailed Wilcoxson Sign-Rank Test (= Wilcoxson) evaluating heterozygosity excess and deficiency in 6 northern bobwhite quail populations from Illinois (N=434).** Data derived from 11 microsatellite (msat) DNA loci. Counties = Marion (MAR), Saline (SAL), Washington (WSH), Wayne (WAY), Scott (SCO), Perry (PER); N = Sample size; All pairwise comparisons were significant save for that indicated in bold text and with an asterisk. Mode = Presence of an L-shaped allele frequency distribution (expected under a mutation-drift equilibrium model). $N_e$ = effective population size. 95% CI = Upper and lower confidence intervals for $N_e$. Data from Berkman et al. (2012).

| Counties | N | Wilcoxson | Mode | $N_e$ | 95% CI |
|---|---|---|---|---|---|
| MAR | 122 | 0.003 | OK | 107 | 129-91 |
| SAL | 137 | 0.012 | OK | 63 | 72-56 |
| WSH | 78 | 0.007 | OK | 63 | 76-52 |
| WAY | 52 | 0.016 | OK | 53 | 72-42 |
| SCO | 23 | **0.278*** | OK | 56 | 120-35 |
| PER | 22 | 0.047 | OK | 31 | 50-22 |
